# Effects of cotrimoxazole prophylaxis on *Talaromyces marneffei* infection in HIV/AIDS patients receiving antiretroviral therapy: a retrospective cohort study

**DOI:** 10.1080/22221751.2019.1588078

**Published:** 2019-03-11

**Authors:** Junjun Jiang, Fengxiang Qin, Sirun Meng, Eric J. Nehl, Jinping Huang, Yanfen Liu, Jun Zou, Wenyi Dong, Jiegang Huang, Hui Chen, Ning Zang, Bingyu Liang, Chuanyi Ning, Yanyan Liao, Chaolian Luo, Huifang Liu, Xin Liu, Jian Wang, Oulu Zhou, Thuy Le, Li Ye, Fengyao Wu, Hao Liang

**Affiliations:** a Guangxi Key Laboratory of AIDS Prevention and Treatment & Guangxi Universities Key Laboratory of Prevention and Control of Highly Prevalent Disease, School of Public Health, Guangxi Medical University, Nanning, People’s Republic of China; b Fourth People’s Hospital of Nanning, Nanning, People’s Republic of China; c Rollins School of Public Health, Emory University, Atlanta, GA, USA; d Guangxi Collaborative Innovation Center for Biomedicine, Life Sciences Institute, Guangxi Medical University, Nanning, People’s Republic of China; e Division of Infectious Diseases, Duke University School of Medicine, Durham, NC, USA; f Oxford University Clinical Research Unit, Ho Chi Minh City, Vietnam

**Keywords:** HIV, Talaromyces marneffei, cotrimoxazole, antiretroviral therapy, AIDS

## Abstract

The dimorphic fungus *Talaromyces marneffei* (TM) is a common cause of HIV-associated opportunistic infections in Southeast Asia. Cotrimoxazole (CTX) inhibits folic acid synthesis which is important for the survival of many bacteria, protozoa, and fungi and has been used to prevent several opportunistic infections among HIV/AIDS patients. We question whether CTX is effective in preventing TM infection. To investigate this question, we conducted an 11-year (2005–2016) retrospective observational cohort study of all patients on the Chinese national antiretroviral therapy (ART) programme in Guangxi, a province with high HIV and TM burden in China. Survival analysis was conducted to investigate TM cumulative incidence, and Cox regression and propensity score matching (PSM) were used to evaluate the effect of CTX on TM incidence. Of the 3359 eligible individuals contributing 10,504.66 person-years of follow-up, 81.81% received CTX within 6 months after ART initiation, and 4.73% developed TM infection, contributing 15.14/1,000 person-year TM incidence rate. CTX patients had a significantly lower incidence of TM infection than non-CTX patients (4.11% vs. 7.53%; adjusted hazard ratio (aHR) = 0.50, 95% CI 0.35–0.73). CTX reduced TM incidence in all CD4^+^ cell subgroups (<50 cells/μL, 50–99 cells/μL, 100–199 cells/μL), with the highest reduction observed in patients with a baseline CD4^+^ cell count <50 cells/μL in both Cox regression and the PSM analyses. In conclusion, in addition to preventing other HIV-associated opportunistic infections, CTX prophylaxis has the potential to prevent TM infection in HIV/AIDS patients receiving ART.

## Introduction

The human immunodeficiency virus (HIV) causes Acquired Immune Deficiency Syndrome (AIDS) which puts people at risk for opportunistic infections from pathogens that rarely affect healthy people, including *Mycobacterium tuberculosis* (MTB), *Talaromyces marneffei* (TM, previously known as *Penicillium marneffei*), Human Herpes Virus-8 (HHV-8, which causes Kaposi’s sarcoma), and *Pneumocystis jiroveci* (which causes *Pneumocystis joroveci* pneumonia, PJP) [1]. Antiretroviral therapy (ART) suppresses HIV replication, restores immune function, and reduces the risk of opportunistic infections [2]. As a result global AIDS-related deaths have declined by 43% since 2003 [3,4].

As of July 2017, more than 900,000 individuals in China were living with HIV or AIDS [5], with tuberculosis and invasive fungal infections being the major causes of death [6]. TM infection has rapidly emerged in parallel with the HIV epidemic in recent years [7] and has become one of the most common HIV-associated opportunistic infections and is a leading cause of HIV-associated death in Southeast Asia, in particular Thailand, Vietnam, Hong Kong, and southern provinces in China [7–13]. The mortality is up to 30% on antifungal therapy and is up to 100% with delayed diagnosis and treatment [10,13]. Approximately 50,000 HIV/AIDS patients are estimated to be infected with TM each year, with over 5,000 deaths being attributed to TM annually [10,14]. In China, the majority of TM infections have been reported from the Guangxi and Guangdong provinces, with 90% of cases being newly or previously diagnosed with HIV infection[10,15,16]. In these two southern provinces in China, TM infection in HIV/AIDS patients has become a major medical and public health problem, which urgently requires novel preventive and control strategies.

Cotrimoxazole (CTX), a combination of trimethoprim and sulfamethoxazole, is widely used as a primary prophylaxis treatment against several fungal and protozoa infections in patients with advanced HIV disease including PJP, malaria, and cerebral toxoplasmosis [17–19]. In addition to reducing morbidity and mortality associated with PJP and toxoplasmosis, CTX has been shown to reduce the morbidity and mortality associated with malaria and diarrhea in Africa [19]. CTX has also been shown to reduce the risk of tuberculosis among both ART-naïve and ART-experienced children in an African study and in the Swiss HIV cohort study [20,21]. In the Swiss cohort study, CTX use was independently associated with reduced all-cause mortality following adjustment for several variables including ART [21]. An observational cohort study in China also showed that CTX treatment started early during ART could reduce mortality of adult HIV-infected patients [22]. Since the antimicrobial effect of CTX comes from its blocking activity of folic acid biosynthesis pathway which are important for the survival of many bacteria, protozoa, and fungi [23], we hypothesize that CTX might have protective effect agains TM infection.

China’s national free antiretroviral treatment programme (NFATP) was launched in 2003. Two years later, China began to recommend CTX prophylaxis for all HIV patients older than 14 years who had a CD4^+^ cell count <200 cells/μL [22]. Guangxi ranks second in the burden of HIV disease and has the highest number of TM cases in China [10,24]. The Fourth People’s Hospital of Nanning (FPHN) in Guangxi is the largest HIV treatment centre in Guangxi’s capital city Nanning, providing ART to over 8,000 patients. In our recent analysis of 1093 TM patients at FPHN between 2012 and 2015, TM infection accounted for 16.1% of HIV admission and had the highest in-hospital mortality (17.5%) among HIV-associated complications including tuberculosis and cryptococcal meningitis [25]. In this study, we retrospectively collected data from the Chinese NFATP database and the electronic medical records from the FPHN with the aim of investigating the effect of CTX prophylaxis on TM infection among people receiving ART in Guangxi.

## Results

### Demographic characteristics of eligible HIV/AIDS patients at baseline

**Figure 1.**
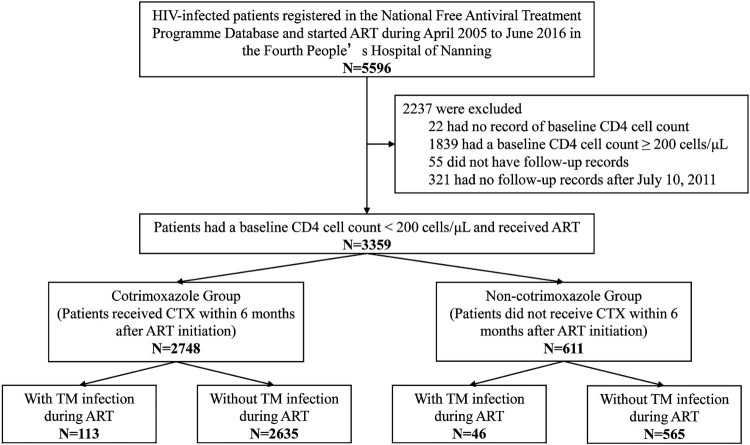
Patient enrolment flowchart.

A total of 5,596 HIV/AIDS patients who initiated ART between April 2005 and June 2016 at the FPHN were screened for inclusion in the present study. Of these, 2,237 patients were excluded, including 22 missing baseline CD4^+^ cell count, 1,839 with a CD4^+^ cell ≥200 cells/μL, 55 missing follow-up record, and 321 missing follow-up record after 10 July 2011. Ultimately, 3,359 patients met the inclusion criteria. Of them, 2,748 received CTX (CTX group) and 611 did not receive CTX (non-CTX group) within 6 months after ART initiation (Figure 1).

Totally, the 3,359 eligible patients contributed 10,504.66 person-years of follow-up, with a median of 2.47 person-year (IQR 0.82–5.07). Table 1 shows the demographic characteristics of the eligible HIV/AIDS patients at baseline. The median age was 42.0 years (IQR 33.00–54.00). 75.02% were male; 69.54% were married or cohabiting, and 91.16% acquired HIV through sexual transmission. The median baseline CD4^+^ cell count at ART initiation was 46.00 cells/μL (IQR 20.00–110.00) and the baseline body mass index (BMI) was 19.53 kg/m^2^ (IQR 17.72–21.48). More than half of these individuals (51.53%) had World Health Organization (WHO) HIV clinical stage IV disease. The majority of patients (92.53%) were treated with nucleotide reverse transcriptase inhibitors (NRTI) combined with non-nucleotide reverse transcriptase inhibitors (NNRTI) as the initial ART regimen. The mean time from clinic registration to initiation of ART was 1.00 month (IQR 1.00–3.00).

**Table 1.** Characteristics of HIV-infected patients receiving ART, by cotrimoxazole prophylaxis.

Variable	Total *n* (%)	Non-CTX group *n* (%)	CTX group *n* (%)	*χ*^2^/Z	*p*
Age at ART initiation				9.924	0.042
<30	502 (14.94)	102 (16.69)	400 (14.56)		
30–39	1,005 (29.92)	182 (29.79)	823 (29.95)		
40–49	766 (22.80)	124 (20.29)	642 (23.36)		
50–59	571 (17.00)	91 (14.89)	480 (17.47)		
≥60	515 (15.33)	112 (18.33)	403 (14.67)		
Gender				11.198	0.001
Male	2,520 (75.02)	426 (69.72)	2,094 (76.20)		
Female	839 (24.98)	185 (30.28)	654 (23.80)		
Marital status				0.348	0.931
Single, divorced or widowed	1,019 (30.34)	183 (29.95)	836 (30.42)		
Married or cohabitation	2,336 (69.54)	428 (70.05)	1,908 (69.43)		
Unknown	4 (0.12)	0	4 (0.15)		
Route of HIV transmission				11.08	0.004
Blood or plasma transfusion	232 (6.91)	53 (8.67)	179 (6.51)		
Sexual transmission	3,062 (91.16)	538 (88.05)	2,524 (91.85)		
Other/unknown	65 (1.94)	20 (3.27)	45 (1.64)		
Baseline CD4 cell count (cells/μL)				106.83	<0.001
<50	1,755 (52.25)	228 (37.32)	1,527 (55.57)		
50–99	658 (19.59)	109 (17.84)	549 (19.98)		
100–199	946 (28.16)	274 (44.84)	672 (24.45)		
WHO clinical stage				156.7	<0.001
I	628 (18.70)	201 (32.90)	427 (15.54)		
II	335 (9.97)	62 (10.15)	273 (9.93)		
III	665 (19.80)	159 (26.02)	506 (18.41)		
IV	1,731 (51.53)	189 (30.93)	1,542 (56.11)		
Baseline body mass index, kg/m^2^				6.764	0.034
<18.5	1,015 (34.90)	180 (31.49)	835 (35.74)		
18.5–23.9	1,652 (56.81)	332 (58.04)	1,320 (56.51)		
≥24.0	241 (8.29)	60 (10.49)	181 (7.75)		
Initial ART regimen				51.284	<0.001
NRTI + NNRTI	3,108 (92.53)	525 (85.92)	2,583 (94.00)		
NRTI + PI	229 (6.82)	82 (13.42)	147 (5.35)		
Others	22 (0.65)	4 (0.65)	18 (0.66)		
Duration of follow-up (years)*	2.47 (0.82–5.07)	2.40 (1.09–3.49)	2.49 (0.78–5.44)	−3.43	0.001
Delayed time to ART initiation (months)*	1.00 (1.00–3.00)	1.00 (1.00–5.00)	1.00 (1.00–2.00)	−4.77	<0.001

*Data are presented as medium ± interquartile range (IQR), and non-parametric tests were used to compare the characteristics between the two groups.

### Cotrimoxazole prophylaxis was associated with lower TM incidence rate

**Figure 2.**
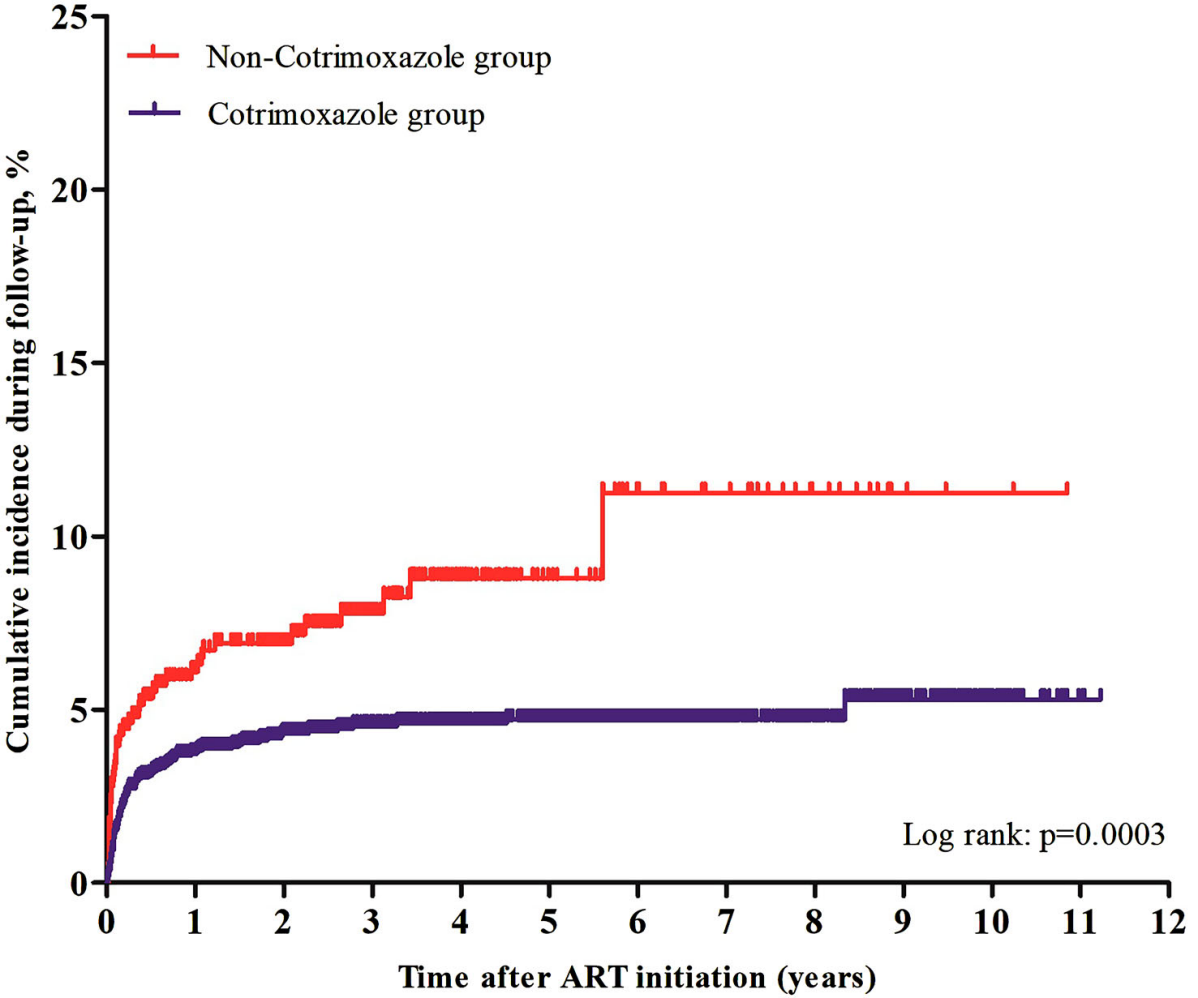
Kaplan-Meier analysis of cumulative incidence of TM infection for HIV/AIDS patients receiving ART, grouped by cotrimoxazole prophylaxis. The statistical significance was measured by log-rank test.

Overall, 4.73% (159 of 3,359) developed a TM infection over a median follow up period of 2.47 years, which contributed to a TM incidence rate of 15.14/1,000 person-year (95% CI 12.84–17.43). In the CTX group, 4.11% (113 of 2748) developed TM infection, which contributed to a TM incidence rate of 12.63/1,000 person-year (95% CI 10.36–14.89). In the non-CTX group, 7.53% (46 of 611) developed TM infection, which contributed to a TM incidence rate of 29.59/1,000 person-year (95% CI 21.26–37.93) (Table 2). Both Chi-squared test (*p* < 0.001) and log-rank test (*p* = 0.0003) showed that the TM incidence rates were significantly different between the CTX and non-CTX groups (Table 2). The Kaplan-Meier chart showed that the CTX group had a significant lower TM cumulative incidence than the non-CTX group over the 11-year follow up period (log-rank test: *p* = 0.0003, Figure 2).

**Table 2.** Comparison of TM infection rate of HIV/AIDS patients with or without Cotrimoxazole prophylaxis.

Group	Total patients *n*	TM infection *n* (%)	*χ*^2^	p*	Person-year	TM infection/1,000 Person-year (95%CI)	p**
CTX	2,748	113 (4.11%)			8950.27	12.63 (10.36–14.89)	
Non-CTX	611	46 (7.53%)			1554.39	29.59 (21.26–37.93)	
Total	3,359	159 (4.73%)	12.94	<0.001	10504.66	15.14 (12.84–17.43)	0.0003

*p by Chi-squared test.

**p by log-rank test

### Cotrimoxazole prophylaxis and baseline CD4^+^ cell count were two major factors affecting TM infection

Cox regression analysis was used to identify the factors that affect TM incidence (Table S1). As shown in Table 3, univariate analyses demonstrated that TM incidence was significantly lower in CTX group compared with those in non-CTX group [hazard ratio (HR) = 0.54, 95% CI 0.38–0.76]. After adjustment for a collection of pre-defined and forward-selection variables (including CTX use, age, gender, marital status, HIV transmission route, WHO HIV disease stage, ART regimen, baseline BMI, baseline CD4 cell count, Hepatitis B surface antigen (HBSAg) positivity, Hepatitis C Virus (HCV) infection, tuberculosis infection in the past year, other opportunistic infections in the past three months including thrush, hairy cell leukoplakia, PJP, esophageal candidiasis, extrapulmonary tuberculosis, and toxoplasmic encephalitis, and delayed time to ART initiation), the CTX group still had a significantly lower TM incidence than the non-CTX group [adjusted hazard ratio (aHR) = 0.50, 95% CI 0.35–0.73, *p* < 0.001] (Table 3).

**Table 3.** Effect of CTX prophylaxis on TM infection among HIV/AIDS patients receiving ART.

CTX prophylaxis	Total patients *n*	TM infection *n* (%)	HR* (95%CI)	p_HR_*	aHR** (95%CI)	p_aHR_**
Yes	2748	113 (4.11%)	0.54(0.38–0.76)	<0.001	0.50(0.35–0.73)	<0.001
No	611	46 (7.53%)	1	–	1	–

*HR: hazard ratio.

**aHR: adjusted hazard ratio, adjusted by CTX use, ART age, gender, marital status, transmission route, initial WHO stage, initial ART regimen, baseline BMI, baseline CD4^+^ cell count, HBV, HCV infection, TB infection in the past year, other opportunistic infections in the past three months (including thrush, hairy leukoplakia, PCP, oesophageal candidiasis, extrapulmonary TB and toxoplasmic encephalitis), and delayed time to ART initiation.

In addition to CTX prophylaxis, a lower CD4^+^ cell count was independently associated with a higher TM incidence (CD4^+^ cell count <50 cells/μL: aHR = 5.83, 95% CI 3.04–11.18, *p* < 0.001; CD4^+^ cell count of 50–99 cells/μL: aHR = 3.43, 95% CI 1.67–7.03, *p* = 0.001; referenced to CD4^+^ cell count of 100–199 cells/μL) (Table S1). As shown in Table 4, the highest TM incidence (14.47%, or incidence rate 60.32/1,000 person-year, 95% CI 40.26–80.39) was found among non-CTX patients with a CD4^+^ cell count <50 cells/μL, which was significantly higher than those in non-CTX patients with a CD4^+^ cell count of 50–99 cells/μL or with a CD4^+^ cell count of 100–199 cells/μL. Similarly, in the CTX group, TM incidence in patients with a CD4^+^ cell count <50 cells/μL (5.83%, or incidence rate 16.79/1,000 person-year, 95% CI 13.39–20.19) was significantly higher than patients in the CTX group with a CD4^+^ cell count of 50–99 cells/μL or with a CD4^+^ cell count of 100–199 cells/μL (Table 4).

**Table 4.** The TM infection rate of HIV/AIDS patients receiving ART, group by CTX prophylaxis and baseline CD4^+^ cell count.

Group		Total patients n	TM infection n (%)	Person-years	TM infection/1,000 Person-year (95%CI)	P*
Non-CTX	CD4^+^ cell count <50 cells/μL	228	33 (14.47)	547.04	60.32 (40.26–80.39)	<0.0001
	CD4^+^ cell count 50–99 cells/μL	109	6 (5.50)	255.79	23.46 (5.16–41.76)	
	CD4^+^ cell count 100–199 cells/μL	274	7 (2.55)	751.56	9.31 (2.59–16.04)	
CTX	CD4^+^ cell count <50 cells/μL	1,527	89 (5.83)	5,301.24	16.79 (13.39–20.19)	<0.0001
	CD4^+^ cell count 50–99 cells/μL	549	20 (3.64)	1,686.91	11.86 (6.79–16.92)	
	CD4^+^ cell count 100–199 cells/μL	672	4 (0.60)	1,962.12	2.04 (0.09–3.99)	
Total		3,359	159 (4.73)	10,504.66	15.14 (12.84–17.43)	

*p by log-rank test.

In addition to CTX prophylaxis and baseline CD4^+^ cell count, patients who were diagnosed with extrapulmonary tuberculosis in the past three months also had a higher incidence of TM infection (aHR = 1.56, 95% CI 1.02–2.40, *p* = 0.04) (Table S1).

In addition, among 159 patients with TM infection, there was no significant difference in the distribution of seasonality of TM infection between CTX group and non-CTX group (*χ*^2 ^= 0.30, *p* = 0.96, Table S2). And there was no significant difference in the mortality between the CTX group and the non-CTX group in the first 6 months of follow up (log-rank *p* = 0.074, Figure S1).

### Relationship between CTX prophylaxis and TM cumulative incidence grouped by baseline CD4^+^ cell count

**Figure 3.**
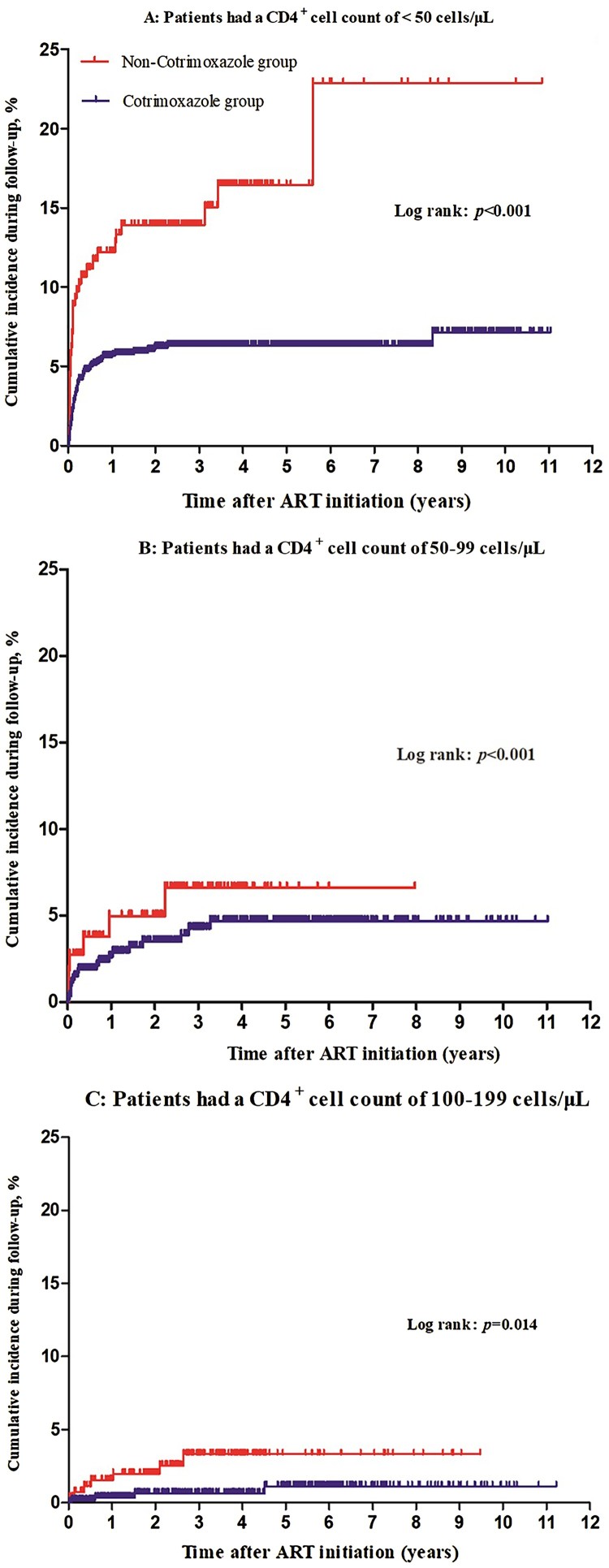
Kaplan-Meier analysis of cumulative incidence of TM infection for HIV/AIDS patients receiving ART, grouped by cotrimoxazole prophylaxis and baseline CD4^+^ cell count. (A) Patients had a baseline CD4 cell count of <50 cells/μL. (B) Patients had a baseline CD4 cell count of 50–99 cells/μL. (C) Patients had a baseline CD4 cell count of 100–199 cells/μL. The statistical significance was measured by log-rank test.

Since baseline CD4^+^ cell count was one major factor influencing TM infection, we evaluated the effect of CTX use on TM infection in three subgroups of CD4^+^ cell count: <50, 50 to 99, and 100–199 cells/μL using Kaplan-Meier method. In all three CD4^+^ cell count subgroups, the cumulative incidences of TM infection in the CTX group were significantly lower than those in the non-CTX group (Figure 3(A–C)). The largest difference in TM cumulative incidence between the CTX and non-CTX groups was found in the subgroup with the lowest CD4^+^ cell count of <50 cells/μL (Figure 3(A)).

### Propensity score matching (PSM) analysis

Since seven variables including age at ART initiation, gender, HIV transmission route, baseline CD4^+^ cell count, baseline WHO HIV clinical stage, baseline BMI, and initial ART regimen were found to be statistically different between the CTX and non-CTX groups in the univariable analyses (Table 1), the distribution of these variables may be different between groups and may introduce biases in the analysis of the CTX effect. To minimize these potential biases, 1:1 PSM was performed. A calliper of 0.0001 was set to ensure all variables were properly matched between the two groups, and a final number of 906 patients (453 CTX and 453 non-CTX patients) were included. Chi-squared test was subsequently performed to evaluate the effectiveness of the PSM. The results showed that these seven variables no longer exhibited statistically significant differences after matching (data not shown). Cox proportional hazard regression was then used to adjust for the effect of CTX treatment on TM infection. The PSM analysis showed that CTX patients had lower risk for TM infection than non-CTX patients (HR = 0.44, 95% CI 0.25–0.77, *p* = 0.004; aHR = 0.52, 95% CI 0.28–0.96, *p* = 0.04) (Table 5). The effect of CTX use on TM infection risk evaluated by the PSM analysis was similar to that in the Cox regression analysis of the whole patient population (aHR = 0.50, 95% CI 0.35–0.73, *p* < 0.001) (Table 3).

**Table 5.** Effect of CTX prophylaxis on TM infection among HIV/AIDS patients receiving ART after propensity score matching.

CTX prophylaxis	Total patients *n*	TM infection *n* (%)	Person-years	TM infection /1,000 person-year (95%CI)	HR* (95%CI)	aHR** (95%CI)
Yes	453	18 (3.97)	1,286.58	13.99 (7.69–20.29)	0.44(0.25–0.77)	0.52(0.28–0.96)
No	453	39 (8.61)	1,011.16	38.57 (26.77–50.37)	1	1

*HR: hazard ratio.

**aHR: adjusted hazard ratio, adjusted by CTX use, ART age, gender, marital status, transmission route, initial WHO stage, initial ART regimen, baseline BMI, baseline CD4 cell count, HBV, HCV infection, TB infection in the past year, other opportunistic infections in the past three months (including thrush, hairy leukoplakia, PCP, oesophageal candidiasis, extrapulmonary TB and toxoplasmic encephalitis), and delayed time to ART initiation.

## Discussion

In this 11 year retrospective cohort study of HIV-infected patients starting ART in Guangxi province in China with a CD4^+^ cell count of <200 cells/µL, we report for the first time a TM incidence and an incidence rate 4.73% over a median follow up period of 2.47 years and 15.14/1,000 person-year, respectively. These numbers are lower than the reported TM prevalence of 16.1% among hospitalized HIV-infected patients in our recent study from the same hospital (FPHN) [25], because these are incidence estimates from an outpatient (and presumably asymptomatic) HIV-infected population with advanced HIV disease. These incidence estimates likely represent a small fraction of true disease burden because our current culture-based diagnosis is suboptimal and is only able to detect disease in its advanced stage. We lack non-culture-based diagnostic modalities to detect disease in its full clinical spectrum. In addition, not all patients who develop disease present to the hospital and receive a diagnosis. Despite these limitations, these incidence estimates are important as they emphasize a substantial burden of diagnosed and undiagnosed TM infection among HIV/AIDS patients with advanced HIV disease on ART in Guangxi and advocate for research to improve disease diagnosis and prevention strategies.

Our study revealed that CTX prophylaxis starting within 6 months after ART initiation was associated with a reduced incidence of TM infection in patients with a CD4^+^ cell count of <200 cells/µL. This finding is supported by both the whole population multivariable Cox regression analysis of a relatively large study population with fairly good follow up data and by the PSM sub-population analysis that sought to reduce biases in the distribution of potentially confounding variables. Our finding is novel and interesting; however, there are some potential factors that might influence the finding. The first factor is the baseline CD4^+^ cell count. Lower CD4^+^ cell counts have been associated with opportunistic infections such as tuberculosis and PJP among HIV/AIDS patients [26–28]. Several studies have also confirmed an association of TM infection with a lower CD4^+^ cell count [10,29]. In our study, the baseline CD4^+^ cell count was identified to be a major factor influencing TM infection (Table S1). Was there a significant difference in baseline CD4^+^ cell count between CTX group and non-CTX group? To address this issue, Kaplan-Meier curves of TM accumulated incidence were drawn for each CD4^+^ cell subgroup and a PSM analysis matched for baseline CD4^+^ cell count was performed. In all three CD4^+^ cell count subgroups and in the PSM analysis with matching baseline CD4^+^ cell count, the association between CTX use and reduced TM infection was consistently observed, indicating baseline CD4^+^ cell count does not affect the relationship of CTX use and reduced TM infection. In fact, the benefit of CTX was most significant in the lowest CD4^+^ cell count subgroup (<50 cells/µL). This provides further support for the finding because if CTX has an inhibitory effect on TM infection, the effect would be expected to be most profound in the patient group who has the highest burden of TM infection, and this was what we found. Another potential confounder is the difference in risk of TM exposure of patients. TM infection is known to occur more frequently during the rainy months in northern Thailand and southern Vietnam [13,30]. Environmental factors such as precipitation and humidity have shown to impact on occurrence of TM infection [13,31]. These environmental factors were not measured for each patient in this study due to the inherent deficiency of our retrospective study design. Although a thorough evaluation of all environmental factors affecting TM incidence is not feasible for this study, we have addressed this issue by evaluating the distribution of the seasons when the TM cases were diagnosed for the CTX and non-CTX groups, and we found that the distributions of seasonality of TM diagnoses between CTX group and non-CTX group were not statistically different (Table S2), indicating that the seasonality does not affect the relationship of CTX use and reduced TM infection. In addition, we also found that the mortality between the CTX group and the non-CTX group in the first 6 months of follow up was similar (Figure S1), which means that early mortality was not an explanation for the reduced TM infection in the CTX group.

In addition to the potential confounders discussed above, the higher incidence of TM infection in the non-CTX group can also be explained by the potential reasons why these patients were not on CTX in the first place. It is possible that these patients were more marginalized, more mobile, and more difficult to treat due to socioeconomic factors that were not measured in this study. In addition, although all patients received ART in this study and time to ART initiation was not different between the groups, ART adherence was not measured. Non-CTX patients could have lower levels of ART adherence, leading to less effective ART and higher risks for TM infection and other opportunistic infections. Poor ART adherence and ART ineffectiveness can also explain an interesting jump of TM incidence in the non-CTX group in the later years (after 6 years on ART) as we do not expect the benefit of CTX to persist that long if ART was effective (Figure 3(A)). The jump in TM cases during the later years was only observed in patients with a baseline CD4^+^ cell count <50 cells/μL (Figure 3(A)), again supporting a hypothesis that these patients were relatively more marginalized and more vulnerable population. However, our data cannot rule out the possibility of a long-term benefit of early CTX prophylaxis in TM prevention.

Not withstanding inherent limitations of a retrospective study, our study provides the first epidemiological observation for the preventive effect of CTX on TM infection but does not prove causality. CTX is a well-known antimicrobial agen used to treat many bacterial infections but also has activities against protozoa, parasitic, and fungal infections such as toxoplasmosis, malaria, and PJP [32]. Its antimicrobial effect comes from its blocking activity of the folate (vitamin B9) biosynthesis, which is an intrinsic mechanism of bacteria and crucial for bacterial survival [23]. The folate biosynthesis doesn’t exist in the human body. However, many fungi in the fungal kingdom including TM also possess folate biosynthesis pathway, which has been highlighted as an antifungal target for the development of new antifungals [23,33]. Since CTX has inhibitory effect on folate biosynthesis, it may exhibit antifungal function, as in the case against PJP [34]. Therefore, CTX could inhibit TM infection by interfering with folate biosynthesis pathway of TM. However, due to unmeasured confounding factors mentioned above, further epidemiological studies are needed to confirm our findings, and *in vitro* and *in vivo* experiments testing the antifungal activities of CTX against TM and other fungi should be conducted to examine the causality of the protective effect of CTX on TM infection.

## Conclusions

In our large 11-year retrospective cohort study, we estimated for the first time the incidence of TM infection in patients with advanced HIV disease starting ART in southern China. We also showed for the first time a protective effect of CTX prophylaxis on TM infection in this population, and showed that protective effect was more profound in patients with a CD4^+^ cell counts <50 cells/μL. As countries or regions suffering from a TM epidemic, such as Guangxi, are in urgent need to decrease the burden of TM infection, we advocate for further studies to determine whether CTX prophylaxis might be considered as an effective intervention against TM infection among patients with advanced HIV disease in China and Southeast Asia.

## Materials and methods

### Study site and population

This retrospective cohort study was conducted at the FPHN, which is the largest specialized infectious disease tertiary hospital and HIV/AIDS treatment centre in Guangxi, China. All HIV-positive individuals with standard ART from FPHN were reported to the NFATP, and recommended for hospitalization following diagnosis with TM infection. The Chinese NFATP database does not recognize TM as a distinct disease but groups it in the category of disseminated fungal diseases. However, the NFATP database is linked with the electronic medical records from the FPHN, which allows for a differentiation among the invasive fungal infections including cryptococcal meningitis, talaromycosis, PJP, and esophageal candidiasis. We retrospectively collected data from April 2005 to June 2016 for all HIV/AIDS patients who were older than 16 years who started ART during this study period. Patients were excluded if they met the following criteria: (i) had no baseline CD4^+^ cell count, (ii) had a baseline CD4^+^ cell count ≥200 cells/μL (i.e. patients who would not be qualified for CTX prophylaxis), (iii) had no follow-up records, (iv) had no follow-up records after 10 July 2011 (because the FPHN started to record TM infection as a separate disease after that date). Data from eligible individuals were collected using a standardized case report form. Data collected included baseline CD4^+^ cell count, WHO HIV clinical stage, time from clinic registration to ART initiation, seasonality at time of TM infection (Spring, Summer, Fall, Winter), Hepatitis B surface antigen (HBSAg) positivity, Hepatitis C Virus (HCV) chronic infection (i.e. positive HCV RNA), thrush, hairy cell leukoplakia, PJP, esophageal candidiasis, extrapulmonary tuberculosis, and toxoplasma encephalitis infection [13,16,30]. The time of TM diagnosis was obtained from the electronic medical records from the FPHN. The study was approved by the Human Research Ethics Committee of Guangxi Medical University. The identity and information of all participants were stored in the hospital’s medical record system, which a secure password protected system that is not open to the public or unauthorized users.

### Definitions

CTX prophylaxis receipt was recorded in the NFATP database at each follow-up visit following ART initiation. Since the exact date on which CTX treatment was started or stopped was not recorded, we regarded patients who received CTX within 6 months after ART initiation as the CTX group and patients who did not received CTX use (i.e. no documentation of CTX use was recorded) within 6 months after ART initiation as the non-CTX group. The observation period was from the date of ART initiation to the date of TM diagnosis. TM diagnosis was confirmed by positive fungal culture from any sterile body site such as blood, bone marrow, lymph nodes, skin lesions, or other body fluids; while non-TM-infected patients included those who were uninfected or asymptomatic. In addition, time to death, time to loss of follow-up, or time to the last follow-up visit before June 2016 were recorded. Baseline was defined as various demographic and clinical indices from the most recent record prior to or following ART initiation.

### Statistical analysis

The selection of independent variables was based on the results of previous studies indicating factors that are associated with TM infection, including antiretroviral therapy (ART), CD4 cell counts, WHO HIV clinical stages, HIV transmission routes, tuberculosis, and other opportunistic infections, etc. Categorical variables were expressed as a frequency or proportion, while quantitative variables were expressed as the median ± interquartile range (IQR). Chi-squared (for categorical variables) and non-parametric (for quantitative variables) tests were used to compare the characteristics between the two groups. Kaplan-Meier graph was used to show the accumulative TM infection during the follow up period. The statistical testing of difference was performed using the log-rank test. Univariable and multivariable Cox regression analyses were used to evaluate the effect of CTX on TM incidence.

For the 1:1 propensity score matching, the characteristics that were statistically different between CTX and non-CTX groups were marched using a calliper beginning with 0.02, and all variables were matched at a difference of the logit of the propensity score of 0.0001 SD. A Chi-squared test was then performed to evaluate the effectiveness of the propensity score matching. Subsequently, a multivariable Cox proportional hazard regression was used to investigate the independent effect of CTX treatment on TM infection.

Data were analyzed using the Statistical Package for the Social Sciences (SPSS) version 23.0 (SPSS Inc. Chicago, USA) and GraphPad Prism version 6.0 (GraphPad Software, San Diego, California, USA). A two-tailed statistical test with a *p* value of 0.05 or less was considered statistically significant.

## Data Availability

All data included in this study are available upon request by contact with the corresponding author.
